# Interventions for improving treatment adherence in young people with inflammatory bowel disease (IBD): A systematic review of behaviour change theory and behaviour change techniques

**DOI:** 10.1177/13674935241310893

**Published:** 2024-12-20

**Authors:** Cassandra Screti, Lou Atkinson, Rachel Shaw, Rafeeq Muhammed, Gemma Heath

**Affiliations:** 1 1722 School of Health and Life Sciences, Aston University, Birmingham, UK; 2 1729 Gastroenterology and Nutrition, Birmingham Women’s, and Children’s Hospital, Birmingham, UK

**Keywords:** Adolescent health [N01.400.075], inflammatory bowel diseases [C06.405.469.432], medication adherence [F01.145.488.887.500.600.500], systematic review [V03.850], treatment adherence and compliance [F01.100.150.750]

## Abstract

Treatment adherence is important but challenging for young people with inflammatory bowel disease (IBD). Behavioural interventions may support adherence, leading to improved condition management. This review aimed to evaluate interventions designed to improve treatment adherence in young people (aged 13–18) with IBD and identify their use of behaviour change theory and behaviour change techniques (BCTs). Five databases (PsycInfo, Embase, MEDLINE, Web of Science and Scopus) were searched to identify eligible articles published between 1980 and 2022. Articles were critically appraised using the Mixed Methods Appraisal Tool. Findings were synthesised narratively. Seven articles reporting seven oral medication adherence interventions were included. Study designs included five randomised controlled trials and two single-arm clinical trials. Eleven BCTs were identified across seven articles. No article discussed how an intervention was informed by behaviour change theory. Interventions that included additional family members and/or offered tailored adherence support generally had greater effects, as did interventions including education and goal setting components. Reporting of intervention content was poor, limiting our ability to make concrete recommendations regarding intervention effectiveness, use of behaviour change theory and BCTs. Further research is needed to understand how theory-driven behaviour change interventions can improve treatment adherence in young people with IBD.

## Background

Inflammatory bowel disease (IBD) is a collective term used to describe a group of intestinal conditions including Crohn’s disease and ulcerative colitis. IBD involves inflammation of the digestive system causing abdominal pain, diarrhoea and fatigue (Yu and Rodriguez, 2017). Incidences of paediatric IBD are rising, particularly amongst adolescents (Ye et al., 2020). Approximately 77 per 100,000 2–17-year-olds in the USA and 22 per 100,000 10–16-year-olds in the UK are living with IBD (Pasvol et al., 2020; Ye et al., 2020). To control their condition, young people are required to maintain high levels of medication adherence, as well as changes to lifestyle behaviours (Ananthakrishnan et al., 2022; Hanghoj and Boisen, 2014).

Upon IBD diagnosis or symptom flare up, medical interventions aim to place disease activity into remission, followed by maintenance medication to prevent symptom relapse (Elhag et al., 2022; LeLeiko et al., 2013). IBD medications include immunosuppressants, 5-aminosalicylate acids and biological anti-tumour necrosis factor (Anti-TNF) therapies, all of which can be prescribed independently or in combination (Carroll et al., 2019). Exclusive enteral nutrition is also an effective non-pharmacological treatment used to induce remission in young people with IBD (Carroll et al., 2019; Ruemmele et al., 2014). Young people’s treatment plans are further related to their illness severity and may subsequently change over time (Gumidyala et al., 2017).

In addition to medication, people with IBD are often advised to make lifestyle changes to control their symptoms (Ananthakrishnan et al., 2022). This includes monitoring food intake and making dietary adjustments to ensure foods high in fats and sugar are eaten in moderation (Lamb et al., 2019; Miele et al., 2018). While approximately 20% of paediatric patients have been shown to believe diet is more important than medication for treating IBD (Bramuzzo et al., 2022), older adolescents are more prone to ignoring dietary advice (Vlahou et al., 2008). To improve fatigue and disease management, those with IBD are also recommended to engage in moderate exercise (Legeret et al., 2019). However, adolescents with IBD self-report greater levels of sedentary behaviour and lower levels of physical activity frequency than healthy adolescents (Bourdier et al., 2019; Penagini et al., 2022; Sledzinska et al., 2022). Sleep has further been identified as a significant factor in IBD management (Manhart et al., 2016; Rozich et al., 2020). While all teenagers are recommended to sleep for 8–10 hours per night, older adolescents in general sleep less, go to bed later and experience greater social jetlag than younger adolescents (Gariepy et al., 2020; Hirshkowitz et al., 2015).

Rates of medication non-adherence in adolescents with IBD are reported to be as high as 65–93% (Knowles and Alex, 2020; Spekhorst et al., 2016). Factors influencing non-adherence are often complex and related to poor medication knowledge, low social support, challenges to forming medication routines and peer stigmatisation (Knowles and Alex, 2020).

Theoretically, treatment adherence in young people with IBD is understood as a variable behaviour which can be both intentional and unintentional (Clifford et al., 2003). The Necessity and Concerns Framework (NCF) outlines how an individual’s beliefs about the necessity of their medication compared to concerns about administering the medication can impact their adherence behaviours (Foot et al., 2016). The Theory of Planned Behaviour (Ajzen, 1991) and Social Cognitive Theory (Bandura, 1986) further support our understanding of adherence behaviours and the mechanisms required to facilitate behaviour change. TPB suggests individuals are more likely to intend to be adherent if they hold a positive evaluation of medication taking; believe it is socially preferable to perform the behaviour; and feel in control over performing medication behaviours (Ajzen, 1991). Whereas SCT explains behaviour in terms of goals and actions, both are related to individuals’ beliefs around self-efficacy and action-outcomes (Bandura, 1986). Within a large systematic review, components of TPB (e.g., perceived control beliefs) and SCT (e.g., self-efficacy) were shown to predict medication adherence to a wide range of chronic health conditions (Holmes et al., 2014).

Underpinning interventions with health behaviour theory can enhance replicability and support implementation in other settings. Moreover, for effective implementation, behaviour change techniques (BCTs) need to be explicitly specified to support fidelity during delivery (Michie et al., 2013, 2015). Despite an emerging understanding of the barriers young people with IBD face in adhering to their treatment plan, little is known about which behaviour change theories have informed effective interventions, or which BCTs are used within such interventions. Gaining insight into this will optimise future adherence interventions for young people with IBD.

### Aims

This review has three aims: (1) to identify and evaluate treatment adherence interventions for young people with IBD; (2) to identify behaviour change theories underpinning interventions; (3) to identify BCTs used within interventions.

## Method

### Protocol and registration

The review protocol was registered with International Prospective Register of Systematic Reviews (PROSPERO) (registration number: CRD42020158961) and can be accessed through the PROSPERO website (https://www.crd.york.ac.uk/prospero).

### Eligibility criteria

Behavioural interventions with the primary outcome of improving treatment adherence in young people (aged 13–18), with a clinical diagnosis of IBD, in any setting, worldwide, were included. An age range of 13–18 years was selected. This age range is a key milestone in young people’s IBD care, representing a period of transition to adulthood that necessitates increasing responsibility for self-management (Hait et al., 2006; Krauthammer et al., 2020). Treatment adherence encompassed medication adherence, as well as adherence to lifestyle recommendations related to diet, exercise and sleep. In recognition of the developmental context of adherence in young people, interventions which involved parents/caregivers alongside young people were also included. No study types were excluded, allowing for the inclusion of quantitative, qualitative and mixed methodologies. Articles not available in English were excluded.

### Information sources and search terms

PsycInfo, Embase, Web of Science, MEDLINE and Scopus databases were searched for relevant articles published between 1980 and December 2022. The reference lists of all included studies and relevant reviews were searched. A citation search of included studies was also performed. Relevant grey literature was searched using Google Scholar, ProQuest Dissertations and Theses database, Conference Proceedings and Citation Indices for Science and for Social Science and Humanities available through Web of Science. The search strategy included a combination of free text terms and index terms. Synonyms were combined using OR and concepts were combined using AND (Boolean logic). Search terms were developed using the PICOS tool (Richardson et al., 1995): Population (young people with IBD), Intervention (behaviour change), Comparison (any or none), Outcome (treatment adherence) and Study design (any) (see Supplemental File 1).

### Data selection

After removal of duplicates, CS screened the title and abstract of retrieved articles against the inclusion criteria. In cases where title and abstract met the inclusion criteria, full text articles were obtained and screened by three reviewers. CS screened all articles (100%), LA second-screened 57% and GH 43%. Articles that did not meet the inclusion criteria were excluded.

### Data extraction

A data extraction form was used to obtain information from included articles. Extracted data included participant characteristics, study methodology, use of behaviour change theory, use of BCTs, changes to treatment adherence and reported intervention secondary outcomes (e.g. knowledge and wellbeing). Behaviour change theory was coded by two reviewers (CS and LA) using the Theory Coding Scheme (Michie and Prestwich, 2010), a framework containing 19 yes/no statements to identify and explore use of theory within intervention design. BCTs were coded using the Behaviour Change Technique Taxonomy (BCTTv1), a taxonomy of 93 hierarchically clustered BCTs (with definitions), grouped into 16 categories (Michie et al., 2013). The BCTTv1 was applied to intervention descriptions by two reviewers (CS and LA), to identify BCTs. Inter-rater reliability was recorded for the coding of BCTs, aiming for 90% agreement. Following analysis, an almost perfect agreement between the reviewers was found (κ = 0.91; 95% CI [0.84, 0.98]).

### Quality assessment

Included articles were quality appraised using a Mixed Methods Appraisal Tool (MMAT; Pluye et al., 2009). MMAT supports appraisal of methodological quality for quantitative, qualitative and mixed methodological designs (subdivided into three sub-domains: randomised controlled, non-randomised and descriptive). Two researchers (CS and LA) independently assessed the included studies for quality. Only one discrepancy was identified which was resolved through discussion with a third reviewer (GH).

### Data analysis and synthesis

Due to sample size and heterogeneity in methods, meta-analysis was not possible. Narrative synthesis (Popay et al., 2006) was therefore used to summarise extracted data on intervention characteristics, primary and secondary outcomes, use of theory and identified BCTs.

## Results

### Searches

**Figure 1.**
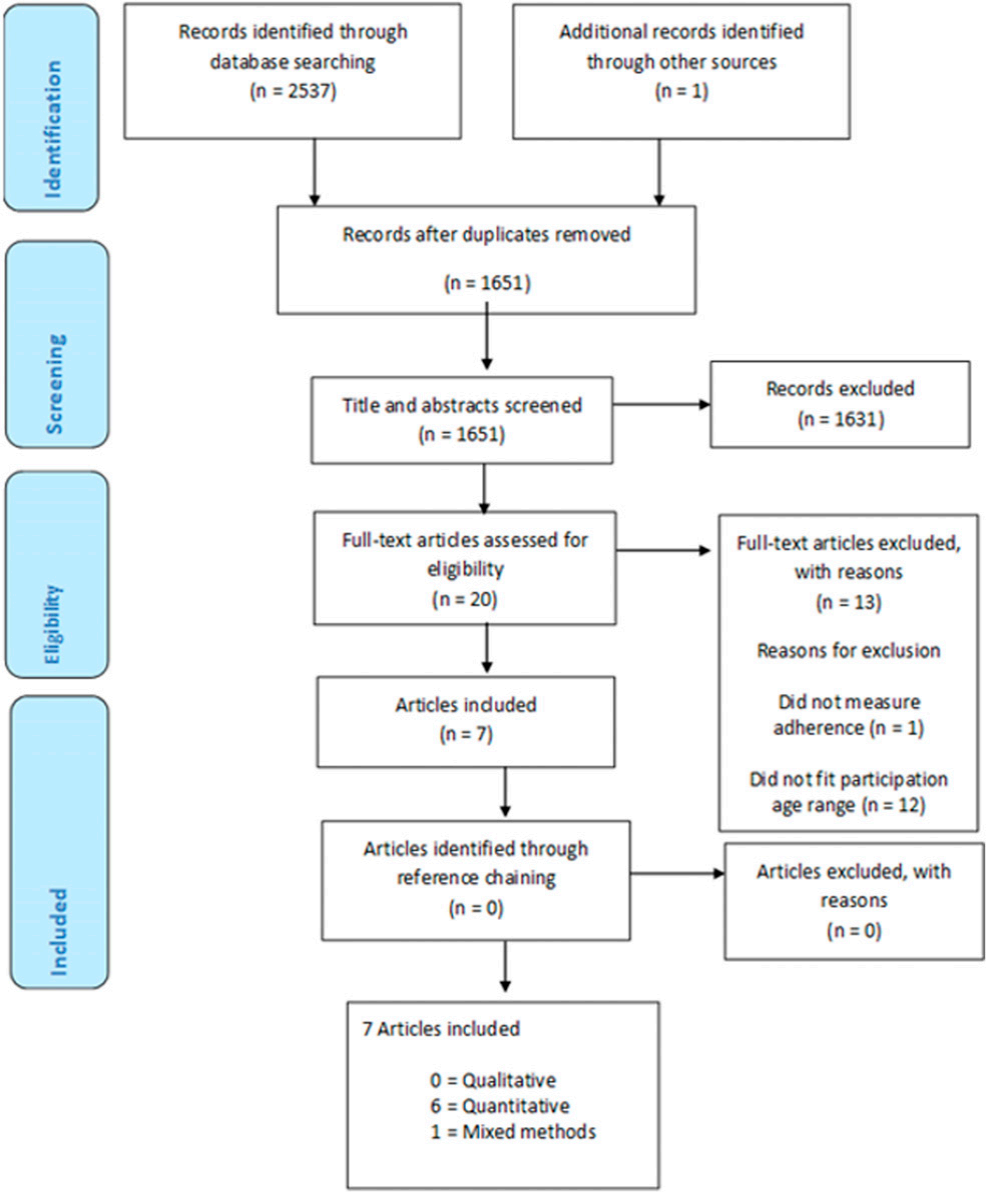
Full list of search terms used.

Following the removal of duplicates, 1651 articles were identified. Title and abstract screening resulted in the exclusion of 1631 studies and the retention of 20 articles. Full-text articles of the remaining studies were screened by the first author, where further 13 articles were excluded; 12 because of participant age, and one for not measuring adherence. This resulted in a final sample of seven articles reporting seven interventions (see Figure 1 [PRIMSA flowchart]).

### Study characteristics

Included interventions aimed to improve young people’s adherence to oral medications; six used quantitative methods and one had a mixed-methods design. Five were evaluated via randomised control trail (RCT), one via a longitudinal single-site noncurrent multiple baseline design across subjects and the other via single-arm clinical trial. Four interventions were delivered within a hospital setting (Hommel et al., 2011, 2012; Maddux et al., 2017; Vaz et al., 2019), one was provided online (Carlsen et al., 2017) and two were delivered via telephone (Greenley et al., 2015; Hommel et al., 2013). Six interventions took place in the USA and one in Denmark (Carlsen et al., 2017). Only RCTs employed control groups, three of which received usual care (Carlsen et al., 2017; Hommel et al., 2012; Vaz et al., 2019) and two as a wait-list control (Greenley et al., 2015; Hommel et al., 2011). Non-RCT studies used pre-post measures of adherence to assess intervention effectiveness (see Table 1 and Supplemental File 2).

**Table 1.** Narrative summary of intervention design, intervention content, use of objective or subjective measures of adherence and medication adherence outputs.

	RCT (yes/no)	Inclusion of a control group	Intervention content	Objective or subjective measure of adherence	Statistically significant improvements to medication adherence
Carlsen et al. (2017)	Yes	Yes	Online intervention asking young people to monitor their health on an intervention-specific website	Subjective	No
Greenley et al. (2015)	Yes	Yes	Family intervention using problem-solving skills training within two or four sessions	Objective	Only for a small subsection of participants, however, the study was not sufficiently powered for a subgroup analysis
Hommel et al. (2011)	Yes	Yes	Four weekly face-to-face group educational sessions	Objective	Only for participants prescribed immunosuppressants
Hommel et al. (2012)	Yes	Yes	Four weekly educational group sessions	Objective and subjective	Only in subjective measures of adherence to mesalamine
Hommel et al. (2013)	No	No	Four weekly educational sessions conducted over the phone	Objective	No
Maddux et al. (2017)	No	No	Four tailored weekly educational sessions	Objective	Yes
Vaz et al. (2019)	Yes	Yes	Single 30-min educational session	Objective and subjective	No

### Participant characteristics

Included studies had a total of 217 young people diagnosed with IBD. Of these, 132 (60.8%) were assigned to receive an intervention, with the remaining 85 (39.2%) assigned to a control condition. Sample sizes varied, ranging from 9 to 76, with an average of 31 young people. The average baseline age of participants across interventions was 14.75 years. There was an even gender split, with 109 participants identifying as female (50.2%). Reporting of ethnicity was poor across all interventions.

The most frequently recorded IBD diagnosis was Crohn’s disease (*n* = 139, 64.1%), a further third was diagnosed with ulcerative colitis (*n* = 73, 33.6%) and the remaining participants were diagnosed with IBD-unknown (*n* = 5, 2.3%). Six of the seven interventions measured participant illness severity via self-report (Carlsen et al., 2017; Greenley et al., 2015; Hommel et al., 2011, 2012, 2013; Maddux et al., 2017). Most participants reported inactive disease (*n* = 110, 53.9%). All interventions required participants to be prescribed at least one oral IBD medication; however, only three studies reported participants’ prescribed medication in detail (Carlsen et al., 2017; Greenley et al., 2015; Hommel et al., 2012). Hommel et al. (2013) only reported the number of those prescribed multiple medications. Most frequently prescribed medications were immunosuppressants (*n* = 98, 58%) followed by 5-aminosalicylic acids (*n* = 87, 51.5%) (see Table 2).

**Table 2.** Young people participant characteristics.

		Carlsen et al. (2017)	Greenley et al. (2015)	Hommel et al. (2011)	Hommel et al. (2012)	Hommel et al. (2013)	Maddux et al. (2017)	Vaz et al. (2019)
Total number of participants	Intervention	*n* = 27	*n* = 50	*n* = 7	*n* = 20	*N* = 9	*N* = 12	*n* = 7
	Control	*n* = 26	*n* = 26	*n* = 7	*n* = 20			*n* = 6
Mean age, years (SD)		15.1 (1.82)	14.54 (1.84)	14.89 (2.01)	15.4 (1.5)	13.71 (1.35)	14.9 (2.16)	14.9 (1.9)
Gender split (M/F)	Intervention	(22/28)	(42/34)	(4/10)	(20/20)	(6/3)	(4/8)	(7/6)
Ethnicity	White/Caucasian	Unknown	*n* = 67	*n* = 14	*n* = 36^a^	*n* = 8	*n* = 8	*n* = 7
IBD diagnosis	Crohn’s disease	*n* = 21	*n* = 55	*n* = 11	*n* = 30	*n* = 7	*n* = 6	*n* = 9
	Ulcerative colitis	*n* = 32	*n* = 19	*n* = 3	*n* = 7	*n* = 2	*n* = 6	*n* = 4
	IBD-unknown		*n* = 2		*n* = 3			
Mean time since diagnosis, years (SD)		Intervention: 2.76 (1.79)	3.52(3.16)	Unknown	Unknown	Unknown	Unknown	Unknown
		Control: 2.26 (1.75)						
IBD severity	Inactive	*n* = 30	*n* = 53	*n* = 6	*n* = 12	*n* = 5	*n* = 4	Unknown
	Mild	*n* = 20	*n* = 19	*n* = 7	*n* = 21	*n* = 3	*n* = 3	Unknown
	Moderate	*n* = 2	*n* = 4	*n* = 1	*n* = 2	*n* = 1	*n* = 5	Unknown
	Moderate to severe				*n* = 5^b^			Unknown
	Severe	*n* = 1						Unknown
Type of IBD medication	Immunosuppressants	*n* = 24	*n* = 50	Unknown	*n* = 24	Unknown	Unknown	Unknown
	5-Aminosalicylate acids	*n* = 32	*n* = 34	Unknown	*n* = 21	Unknown	Unknown	Unknown
	Immunosuppressants and 5-aminosalicylate acids	*n* = 9	Unknown	Unknown	*n* = 6	*n* = 4	Unknown	Unknown

^a^Hommel et al. (2012) reports ethnicity as ‘White, not Hispanic origin’.

^b^Hommel et al. (2012) rating of IBD severity ‘moderate-severe’ was only available for young people diagnosed with ulcerative colitis and IBD-Unknown.

Five interventions included parents (Greenley et al., 2015; Hommel et al., 2011, 2012, 2013; Maddux et al., 2017). Reporting of parent demographics was poor (see Supplemental File 3).

### Withdrawal rates

Four studies reported a withdrawal rate of zero (Hommel et al., 2011, 2012, 2013; Maddux et al., 2017). Within the remaining three, 35 young people were reported to have withdrawn from the research, 65.7% of whom were receiving an intervention (*n* = 23). The median (IQR) withdrawal rate was 0 (0–11) participants per intervention. No intervention separately reported demographic characteristics for those who withdrew from the study.

### Quality assessment

All interventions demonstrated use of a clear research question alongside appropriate data collection methods. Both non-randomised studies (Hommel et al., 2013; Maddux et al., 2017) included appropriate outcome measures; however, the reporting of statistical results was inconsistent within outcome data. Equally, only one study accounted for confounders in the design and analysis (Maddux et al., 2017). Neither non-randomised study clearly reported if the intervention was administered as intended, and it was also unclear if the participants were representative of the target population. The remaining studies reported RCTs (Carlsen et al., 2017; Greenley et al., 2015; Hommel et al., 2011, 2012; Vaz et al., 2019). All RCTs described robust randomisation, with intervention and control groups comparable at baseline. All RCTs reported that participants adhered to the assigned intervention. All RCTs reported complete outcome data. None of the RCT intervention arms were blinded to the person conducting the study (e.g., outcome assessors). Vaz et al. (2019) was also categorised as a mixed-methods design, while the design was appropriate, reporting of the qualitative data was poor (see Table 3).

**Table 3.** Quality assessment of interventions aiming to increase medication adherence in 13–18-year-olds with Inflammatory Bowel Disease.

	Carlsen et al. (2017)	Greenley et al. (2015)	Hommel et al. (2011)	Hommel et al. (2012)	Hommel et al. (2013)	Maddux et al. (2017)	Vaz et al. (2019)
2. Randomised controlled trials							
2.1. Is randomisation appropriately performed?	Yes	Yes	Yes	Yes			Can’t tell
2.2. Are the groups comparable at baseline?	Can’t tell	Can’t tell	Can’t tell	Can’t tell			Can’t tell
2.3. Are there complete outcome data?	No	Yes	No	No			No
2.4. Are outcome assessors blinded to the intervention provided?	No	No	No	No			No
2.5. Did the participants adhere to the assigned intervention?	Yes	Yes	Yes	Yes			Yes
3. Non-randomised studies							
3.1. Are the participants representative of the target population?					Yes	Yes	
3.2. Are measurements appropriate regarding both the outcome and intervention (or exposure)?					Yes	Yes	
3.3. Are there complete outcome data?					No	No	
3.4. Are the confounders accounted for in the design and analysis?					No	Yes	
3.5. During the study period, is the intervention administered (or exposure occurred) as intended?					Can’t tell	Can’t tell	
5. Mixed-methods studies							
5.1. Is there an adequate rationale for using a mixed-methods design to address the research question?							Yes
5.2. Are the different components of the study effectively integrated to answer the research question?							Yes
5.3. Are the outputs of the integration of qualitative and quantitative components adequately interpreted?							No
5.4. Are divergences and inconsistencies between quantitative and qualitative results adequately addressed?							No
5.5. Do the different components of the study adhere to the quality criteria of each tradition of the methods involved?							Yes

Completed using the Mixed Methods Appraisal Tool (MMAT; Pluye et al., 2009).

### Primary outcome – Medication adherence

Two interventions targeted participants at an individual level (Carlsen et al., 2017; Vaz et al., 2019), both of which utilised a control group who received ‘usual care’. The first reported a two-year online intervention, where young people were asked to self-monitor adherence. Biomarkers were then used to provide young people with feedback. This intervention did not produce a statistically significant difference in post-intervention adherence between the intervention and control groups (Carlsen et al., 2017). Equally, a single 30-min IBD medication educational session did not improve post-intervention measures of medication adherence (Vaz et al., 2019).

Five interventions included one or more family members (Greenley et al., 2015; Hommel et al., 2011, 2012, 2013; Maddux et al., 2017). Most of these used a similar four-session family education approach covering the following topics: educational and organisation interventions, behaviour modification, problem-solving skills and family functioning (Hommel et al., 2011, 2012, 2013; Maddux et al., 2017). This did not result in a statistically significant impact on young people’s medication adherence when conducted via the telephone (Hommel et al., 2013) but did produce statistically significant post-intervention improvements for young people prescribed immunosuppressants only, compared with the control group (t = 2.72, *p* < .05), when delivered face-to-face (Hommel et al., 2011). By allowing families to identify their own adherence barriers and set tailored goals, odds ratios identified an increase in adherence (number of doses taken divided by those not taken) at baseline to post-intervention (OR = 1.97) and at a one-month follow-up (OR = 1.36; Maddux et al., 2017). Logistic regression models were run to confirm these increases were significantly different to no change in adherence; however, Maddux et al. (2017) omitted to report the 95% CIs. Hommel et al.’s. (2012) facilitation of four educational sessions delivered as part of a family group intervention only demonstrated a statistically significant increase in adherence to self-reported doses of mesalamine post-intervention, compared with the control group (F = 13.32, *p* < .05, d = 0.69). Greenley et al. (2015) delivered a four-session problem-solving skills training (PSST) intervention via telephone, where PSST guided families to overcome their adherence barriers. Greenley et al.’s. (2015) intervention only improved young people’s medication adherence for older adolescents (aged 16–18) who were ≤80% adherent at baseline (t(13) = 2.50, *p* < .05, d = 0.95). However, Greenley et al.’s. (2015) study was not sufficiently powered for a subgroup analysis.

### Adherence measurement strategies

Four interventions only used objective adherence measurement strategies (Greenley et al., 2015; Hommel et al., 2011, 2013; Maddux et al., 2017). Of these, two interventions solely used pill counts to measure adherence. Hommel et al.’s. (2011) four-session intervention delivered face-to-face produced statistically significant results for those prescribed immunosuppressants only post-intervention when compared to controls (t = 2.72, *p* < .05). However, Hommel et al.’s (2013) four-session intervention did not produce statistically significant improvements in adherence. Greenley et al.’s. (2015) intervention used Medication Event Monitoring System (MEMS) track caps to objectively measure adherence. Following a two-session family intervention, only imperfect adherers at baseline, aged 16–18 years, had a statistically significant increase in adherence post-intervention (t(13) = 2.50, *p* < .05, d = 0.95). Maddux et al. (2017) utilised a combination of pill counts and MEMS track cap measures to determine the success of their tailored intervention, reporting a statistically significant increase in young people’s medication adherence (calculated as number of doses taken divided by those not taken) from baseline to post-intervention (OR = 1.97) and at a one-month follow-up (OR = 1.36). Logistic regression confirmed these were significant increases compared to no change. However, 95% CIs were not reported (Maddux et al., 2017).

Both Hommel et al. (2012) and Vaz et al. (2019) used a combination of objective and subjective adherence measures. Despite finding no statistically significant results using objective measures (pill counts and MEMS track caps), Hommel et al.’s. (2012) four-session family group intervention reported statistically significant improvements in self-reported adherence to mesalamine (F = 13.32, *p* < .05, d = 0.69) when measured using a study-specific Treatment Regimen Adherence Questionnaire (TRAQ). However, this was not observed in parent-proxy self-reported adherence (Hommel et al., 2012). Vaz et al.’s. (2019) single-session education intervention did not result in a statistically significant improvement in medication adherence when measured objectively via pill counts. During post-intervention interviews, participants further reported the intervention had not impacted their adherence behaviours (Vaz et al., 2019).

Carlsen et al. (2017) solely used subjective measures of adherence, self-reporting adherence behaviours every month for 2 years using The Medication Adherence Report Scale (MARS) and a study-specific adherence Visual Analogue Scale (VAS); however, this resulted in non-statistically significant increases in medication adherence.

### Secondary outcomes

One intervention additionally aimed to improve young people’s IBD knowledge (Vaz et al., 2019). This single-session educational intervention did not statistically improve young people’s IBD knowledge quantitatively; however in post-intervention interviews, young people did report improvements in their IBD knowledge. Two interventions measured changes in participants’ Quality of Life (QoL). Using PSST to improve medication adherence resulted in a statistically significant increase in young people’s self-reported QoL (t(66) = −2.83, *p* < .006, d = 0.49; Greenley et al., 2015). Carlsen et al.’s. (2017) two-year digital intervention resulted in a non-statistically significant difference between the intervention and control groups’ overall QoL, and the emotional functioning subscale scores were lower in the intervention group (Estimate = −0.003 per day, 95% CI [−0.006, 0.0003]). This statistically significant difference indicates the control group had a greater emotional functioning QoL than those assigned to the intervention (Carlsen et al., 2017). Equally, the intervention resulted in a non-statistically significant impact on young people’s self-reported symptom scores or biological markers nor did it improve young people’s self-reported transition readiness (Carlsen et al., 2017). Despite this, there was a statistically significant reduction in the number of outpatient appointments for the intervention group (intervention: *n* = 85, control: *n* = 185, *p* < .0001) but not hospital admissions. Those receiving the intervention also reported statistically significantly fewer school absences per participant than the control group (intervention: mean = 1.6 days, SEM 0.5, control: mean = 16.5 days, SEM 4.4, *p* < .002; Carlsen et al., 2017).

### Use of behaviour change theory

None of the included studies reported use of behaviour change theory within their intervention formulation or delivery. Interventions were reviewed to not only identify behaviour change theories/models but also to identify theory-relevant constructs and predictors. Only one intervention (Greenley et al., 2015) mentioned the theoretical construct of self-efficacy (Bandura, 1977), referred to in the context of ‘cognition/self-efficacy’ as a potential barrier to medication adherence for young people, with no further explanation provided.

### Use of behaviour change techniques (BCTs)

Eleven BCTs, as defined within the BCTTv1 (Michie et al., 2015), were identified within the seven included interventions, with an average of five BCTs identified per study (ranging from 2 to 7 BCTs per study). BCT coding was not possible for control groups, as no authors provided descriptions of control conditions (see Table 4).

**Table 4.** Overview of Behaviour Change Techniques (BCTs) within the interventions, identified using the Behaviour Change Technique Taxonomy (BCTTv1; Michie et al., 2013).

Behavioural change techniques identified^a^	Carlsen et al. (2017)	Greenley et al. (2015)		Hommel et al. (2011)	Hommel et al. (2012)	Hommel et al. (2013)	Maddux et al. (2017)	Vaz et al. (2019)
		Intervention group 1	Intervention group 2					
Section 1. Goals and planning								
1.1. Goal-setting (behaviour)		X	X	X	X	X	X	
1.2. Problem solving				X	X	X	X	
1.4. Action planning		X	X					
1.8. Behavioural contract				X	X	X	X	
Section 2. Feedback and monitoring								
2.2. Feedback on behaviour		X	X				X	
2.3. Self-monitoring of behaviour				X	X	X	X	
Section 3. Social support^b^								
Section 4. Shaping knowledge								
4.1. Instruction on how to perform the behaviour	X							
Section 5. Natural consequences								
5.1. Information about health consequences	X			X	X	X	X	X
Section 6. Comparison of behaviour^b^								
Section 7. Associations								
7.1. Prompts/cues				X	X	X		X
Section 8. Repetition and substitution^b^								
Section 9. Comparison of outcomes^b^								
Section 10. Reward and threat^b^								
Section 11. Regulation^b^								
Section 12. Antecedents								
12.1. Restructuring the physical environment				X		X		
12.5. Adding objects to the environment							X	
Section 13. Identity^b^								
Section 14. Scheduled consequences^b^								
Section 15. Self-belief^b^								
Section 16. Convert learning^b^								

^a^BCTs could only be identified in the intervention conditions for all seven interventions.

^b^No BCTs were identified in this section of the BCTTv1 (Michie et al., 2013).

### BCTs associated with successful medication adherence

Three BCTs (*1.4. Action planning*, *2.2. Feedback on behaviour* and *12.5. Adding objects to the environment*) were solely associated with successful medication adherence intervention outcomes. *1.4. Action planning* was identified in Greenley et al.’s. (2015) family intervention which statistically significantly improved medication adherence in older adolescents who displayed imperfect adherence (≤80% at baseline). Two interventions demonstrated the use of *2.2. Feedback on behaviour*; both interventions successfully improved young people’s medication adherence for either the entire sample (Maddux et al., 2017) or a very small subsection of participants (Greenley et al., 2015). Only Maddux et al. (2017) used *12.5. Adding objects to the environment* and reported a statistically significant increase in medication adherence for the intervention group.

### BCTs associated with successful and unsuccessful medication adherence

Seven BCTs were identified across interventions which reported successful and unsuccessful medication adherence outcomes: *1.1. Goal-setting (behaviour)*, *1.2. Problem solving*, *1.8. Behavioural contract*, *2.3. Self-monitoring of behaviour*, *5.1. Information about health consequences*, *7.1. Prompts/cues and 12.1. Restructuring the physical environment*.

Although five interventions included *1.1. Goal-setting (behaviour)*, only four interventions were able to demonstrate a significant increase in medication adherence (Greenley et al., 2015; Hommel et al., 2011, 2012; Maddux et al., 2017). However, within these interventions there was variance in how successful they were. Maddux et al.’s. (2017) four-session intervention, where families were provided with tailored advice, demonstrated a significant improvement in adherence for all participants, whereas Greenley et al.’s. (2015) four-session family intervention only improved adherence for a small subsection of participant who were aged 16–18 years, with ≤80% adherence at baseline. Equally, two interventions utilising a four-session family group intervention were only associated with a successful outcome for those prescribed mesalamine (Hommel et al., 2012) or immunosuppressants (Hommel et al., 2011). However, this BCT was not associated with a successful outcome when used in a family intervention conducted over the telephone (Hommel et al., 2013).

Four family interventions, all comprising four intervention sessions, utilised *1.2. Problem solving*; *1.8. Behavioural contract*; *2.3. Self-monitoring of behaviour* (Hommel et al., 2011, 2012, 2013; Maddux et al., 2017). Three of these reported significant improvements in medication adherence to either the entire intervention group (Maddux et al., 2017) or only for participants prescribed mesalamine (Hommel et al., 2012) or immunosuppressants (Hommel et al., 2011). However, when a similar educational intervention was conducted over the phone this was associated with an unsuccessful medication adherence outcome (Hommel et al., 2013) .

All except one (Greenley et al., 2015) of the included interventions utilised *5.1. Information about health consequences*. However, only three interventions significantly increased levels of medication adherence either for the entire intervention group (Maddux et al., 2017) or for a subsection of participants (Hommel et al., 2011, 2012). Despite using a similar four-session intervention, Hommel et al. (2013) did not report a significant increase in adherence. Equally, the inclusion of 5*.1. Information about health consequences* was not associated with improvements in medication adherence for a single-session educational intervention (Vaz et al., 2019) or a two-year online individual intervention (Carlsen et al., 2017), suggesting that while not always successful, this BCT may be more beneficial in family interventions.

Despite being identified in four interventions, the BCT *7.1. Prompts/cues* was only associated with successful adherence outcome for participants prescribed either mesalamine (Hommel et al. (2012) or immunosuppressants (Hommel et al., 2011) in family-based interventions. When this BCT was used in a family intervention delivered over the telephone (Hommel et al., 2013), as well as within a single-session educational intervention (Vaz et al., 2019), this was not associated with a successful medication adherence outcome. Similarly, two interventions included *12.1. Restructuring the physical environment* (Hommel et al., 2011, 2013); however, only Hommel et al. (2011) reported a successful medication adherence outcome for those prescribed immunosuppressants.

### BCTs associated with unsuccessful medication adherence

One BCT (*4.1. Instruction on how to perform the behaviour)* was associated with a non-statistically significant medication adherence outcome (Carlsen et al., 2017).

## Discussion

This systematic review aimed to evaluate treatment adherence interventions for young people with IBD and to identify their use of behaviour change theory and BCTs. Five databases were systematically searched resulting in the inclusion of seven interventions. While just over half the interventions reported statistically significant improvements in treatment adherence, three interventions only reported medication adherence improvements for a subsection of participants (Greenley et al., 2015; Hommel et al., 2011, 2012). Three BCTs were identified within solely effective interventions; however, health behaviour theory was not found to underpin any of the included interventions. Quality of included studies was assessed as good overall, although reporting was generally poor.

Interventions including family members (Greenley et al., 2015; Hommel et al., 2011, 2012; Maddux et al., 2017) were generally more effective for improving medication adherence than those which solely targeted young people (Carlsen et al., 2017; Vaz et al., 2019). Despite this, parents’ role in family interventions, particularly their role in identifying and overcoming adherence barriers, was poorly reported (Greenley et al., 2015; Hommel et al., 2011, 2012, 2013; Maddux et al., 2017). Parental support is crucial in facilitating and maintaining adherence behaviours in children; however, during adolescence, young people are expected to develop independent health self-management skills in preparation for adulthood (Camp-Spivey et al., 2022; Hait et al., 2006; Stollen et al., 2017). Optimising how parents can support adherence during adolescence, as a time of transition to increasing independence, may then, require further consideration (Jayasooriya et al., 2023; Thomsen et al., 2023).

None of the included articles explicitly mentioned if, or indeed how such interventions were informed by behaviour change theory. This highlights a major limitation in the design and reporting of such interventions, although perhaps reflective of health intervention research more broadly (Jackson et al., 2014; Lippke and Ziegelmann, 2008). Use of behaviour change theories/models is vital for increasing understanding of the mechanisms underpinning how, why and to what extent interventions succeed (Skivington et al., 2021). Similarly, while eleven BCTs were identified across the included interventions, none were described using Michie et al.’s. (2013) BCTTv1. This has implications for standardisation of implementing and reproducing the ‘active ingredients’ of interventions (Hoffmann et al., 2014). Guidance has been developed to assist in the effective reporting of behaviour change interventions (Hoffmann et al., 2014).

Interventions often required participants to identify their own adherence barriers and develop plans or set goals to overcome these (Greenley et al., 2015; Hommel et al., 2011, 2012, 2013; Maddux et al., 2017). Within the reporting of these interventions, there was no description of the barriers or plans/goals as identified by participants. Omitting this information limits understanding regarding whether the intervention was successful for reducing all adherence barriers or just a select few. For example, the barrier of forgetting to administer medications may require a different intervention strategy to the barrier of not wanting to take medication in social settings (Jackson et al., 2014). Future interventions should utilise tools to ensure the most appropriate intervention options are used, to obtain the required behaviour change (Michie et al., 2011).

Interventions included in the review only addressed oral medication adherence. However, IBD medication routines are generally broader than just taking pills (Carroll et al., 2019). In addition, no interventions looked to address lifestyle factors (Ananthakrishnan et al., 2022; Hanghoj and Boisen, 2014). However, previous research has identified difficulties young people with IBD face in adhering to non-oral medications (Knowles and Alex, 2020) and lifestyle advice (Penagini et al., 2022; Vlahou et al., 2008). Health behaviour theories such as the NCF (Foot et al., 2016), SCT (Bandura, 1986) or COM-B model (Michie et al., 2011) could assist in the development of more holistic interventions targeting a broader conceptualisation of treatment than oral medication alone (Keefer and Kane, 2016; Park et al., 2021; Screti, 2023).

Previous research has suggested illness duration as a factor in medication adherence, with a greater length of time since diagnosis linked to higher levels of non-adherence behaviour (Platak et al., 2013; Reed-Knight et al., 2011). While illness duration could have influenced intervention success, only two studies reported length of time since participant IBD diagnosis (Carlsen et al., 2017; Greenley et al., 2015). Reporting participant characteristics in detail is crucial for understanding the outcomes of behavioural interventions (Jones et al., 2020).

### Strengths and limitations

This is the first systematic review to investigate the effectiveness of treatment adherence interventions for young people with IBD, including the application of behaviour change theory and BCTs. Using systematic and robust methods, the review highlights several gaps in existing literature, directing objectives for future research. The review is limited, however, by a lack of detail in intervention reporting which impeded in-depth analysis regarding intervention content and use of behaviour change theory. Only a small number of studies were retrieved during the search process, with six out of seven interventions conducted in the USA, primarily by the same research team. While this could reflect focused inclusion/exclusion criteria, it is more likely a reflection of available interventions. Further research is needed in more geographical regions, to further our understanding of young people’s global treatment adherence needs.

In the seven included interventions, a variety of measures were used to determine medication adherence making it difficult to compare outcome data. There is a need for greater consistency in the types of measures used to assess adherence in interventions for young people with IBD.

### Implications for research and practice

This review highlights several implications for research and practice. First, there is a need for better reporting of participant characteristics (e.g. participant age, gender, IBD diagnosis, medication routine and ethnicity). Second, research is needed to explore optimal approaches for involving parents/family members within adherence interventions. Future research should also provide better descriptions of intervention content, including use of behaviour change theory and BCTs, to support understanding of why an intervention was successful or not and to allow for replicability in other settings. Finally, consideration should be given to understanding treatment adherence in broader terms than simply pill-taking (e.g. different types of medication and lifestyle behaviours).

## Conclusion

This review collates and describes interventions designed to support treatment adherence in young people with IBD. Interventions which included the whole family and/or offered tailored advice to reduce personal adherence barriers produced the most promising results. Interventions involving a combination of education and goal setting strategies were also more likely to be successful than those which included education or self-monitoring alone. However, poor reporting of intervention content, participant characteristics and statistical results, and a lack of theoretical underpinning, meant that current evidence is too weak to make concrete recommendations regarding effectiveness. Further research is needed to understand the benefits of theory-driven behavioural change interventions to improve treatment adherence in young people with IBD.

## Supplemental Material

**Supplemental Material -** Interventions for improving treatment adherence in young people with inflammatory bowel disease (IBD). A systematic review of behaviour change theory and behaviour change techniques Supplemental Material for Interventions for improving treatment adherence in young people with inflammatory bowel disease (IBD). A systematic review of behaviour change theory and behaviour change techniques by Cassandra Screti, Lou Atkinson, Rachel Shaw, Rafeeq Muhammed and Gemma Heath in Journal of Child Health Care.
