# Supplementary material for: Interventions for improving treatment adherence in young people with inflammatory bowel disease (IBD): A systematic review of behaviour change theory and behaviour change techniques
Source: J Child Health Care. 2024 Dec 20;30(1):167–87. doi: 10.1177/13674935241310893 (PMC12982571; doi:10.1177/13674935241310893)
Supplement: Supplemental Material - Interventions for improving treatment adherence in young people with inflammatory bowel disease (IBD). A systematic review of behaviour change theory and behaviour change techniques [file sj-pdf-1-chc-10.1177_13674935241310893.pdf]

Interventions for improving treatment adherence in young people with Inflammatory Bowel Disease (IBD). A Systematic Review of behaviour change theory and behaviour change techniques

Supplementary File 1 - Full list of search terms used

(child* OR “young person” OR adolescen* OR “young people” OR teenager* OR parent* OR family OR paediatric OR pediatric* OR mother OR father) AND (intervention OR “behaviour* change” OR “behavior* change” OR RCT OR “randomised control trial” OR “randomized control trial”) AND (“inflammatory bowel disease” OR IBD OR “Crohn’s Disease” OR “Ulcerative Colitis” OR Crohn’s OR Colitis) AND (“treatment adherence” OR “medication adherence” OR “non-adherence” OR “medication compliance” OR diet OR exercise OR “lifestyle change*”)

Supplementary file 2. Full data extraction of the included studies

**Author, (year) Country, study design,**<br><br>**Adheren ce measure**	**Type of intervention,**<br><br>**Sample numbers and**<br><br>**withdrawal rates**	**Intervention content**	**Data Collection**	**Primary outcomes**	**Secondary outcomes**
Carlsen et al. (2017)<br><br>Denmark .<br><br>RCT.	**Individual intervention**<br><br>27 young people took part in the intervention and 26 young people were allocated to the control	**Intervention group:**<br><br>Two-year online intervention. Participants were asked to self-report medication adherence, quality-of-life, contact with	Participants completed subjective self-reported disease severity every month.<br><br>Every three months, participants	**MARS and Adherence VAS***<br><br>Post-intervention, mixed-effects models identified no statistically significant difference in rates of medication adherence	**Disease Severity***<br><br>Mixed-effects modules showed no difference between groups in symptom

Oral medication adherence	group.<br><br>Twelve young people withdrew from the intervention and eight young people withdrew from the control group.	hospitals, and days off school using an online programme.<br><br>Participants also provided faecal and blood samples and received feedback on these results.<br><br>**Control Group:**<br><br>Received usual care	self-reported their medication adherence, using the MARS and an adherence VAS. Participants also self-reported their quality-of-life, contact with hospitals, and days off school.<br><br>Participants provided faecal and blood samples every three months.	between those who undertook the intervention (mean= 23.7 SEM 0.26; 95% CI [23.2, 24.3]) and the control group (mean=23 SEM 0.5; 95% CI [22.0, 23.9]).	scores.<br><br>**Contacts to hospital***<br><br>Wilcoxon sum rank test showed the intervention group had less outpatient visits (n=8) than controls (n=185), this was statistically significant (p<0.001). While there was a tendency for lower hospital admissions in the intervention group (intervention group=2, control =10), this was not statistically significant (p>0.05).<br><br>**School absences***<br><br>The intervention group had fewer days off school

					(mean=1.6, SEM 0.5) than the control group (mean=16.5, SEM 4.4), t-tests revealed this was statistically significant ($p<0.05$)<br><br>**Quality-of-life***<br><br>Mixed-effects modules revealed the only statistically significant difference between the groups was with the subscale emotional functioning. The intervention group scored significantly lower on this subscale compared to controls (Estimate –0.003 per day, 95% CI [0.006, -

					0.0003]).
Greenley et al. (2015)<br><br>United States of America.<br><br>RCT.<br><br>Oral medication adherence	**Family intervention**<br><br>76 families took part in the intervention; 50 were allocated to receive two intervention sessions. Of this group, a further 21 were randomised to receive an additional two sessions. 26 families were allocated to the control group.<br><br>Two young people withdrew from the control condition. Eight young people withdrew from receiving two intervention sessions and an additional young person withdrew from receiving four intervention sessions	**Intervention**<br><br>18-week intervention with two intervention conditions.<br><br>**Two sessions**<br><br>Received two educational sessions, which covered the following information:<br><br>Session 1- 60-90 minutes<br><br>Introduction/education on five core steps to problem solving. Families received personalised feedback on adherence barriers and chose a specific barrier they wished to work on during the intervention. Families were guided to use PSST to overcome the identified barrier.<br><br>Session 2 – 45 minutes<br><br>The family's	A MEMS track cap was used to objectively measure the amount of successfully administered medication doses at baseline and post-intervention.	**MEMS track cap**<br><br>**Two sessions**<br><br>**Entire Sample (N=76)**. Paired-sample t-tests revealed there was a 3% non-statistically significant increase in adherence from baseline to post-intervention ($t(64)=1.25$, $p \geq 0.05$), which had a small effect size ($d=0.22$).<br><br>**Imperfect adherers (n=41)**. Those with perfect adherence (>80% doses taken) were removed from the sample, leaving a subset of participants referred to as imperfect adherers. Paired-sample t-tests revealed there was a non-statistically significant 6% increase in adherence rates from baseline to post-intervention, ($t(40)=1.85$, $p \geq 0.01$), which had a moderate effect size ($d=0.41$). When imperfect adherers were	**Quality-of-life**<br><br>Paired-sample t-tests found receiving two intervention sessions had a statistically significant 4% increase in quality-of-life ($t(66)=-2.83$, $p<0.05$), which had a moderate effect size ($d=0.49$).<br><br>Paired-sample t-tests revealed a marginal non-statistically significant increase in quality-of-life for those receiving four interventional sessions ($t(16)=-1.16$, $p>0.05$), with a

		success in implementing plans formed in previous sessions was reviewed. Families identified an additional barrier and used PSST to overcome the identified barrier.<br><br>**Four intervention sessions**<br><br>In addition to receiving the two sessions outlined above, a group of young people received a further two 45-minute sessions, which covered the same information/format as Session 2.<br><br>**Control group**<br><br>Wait list control		categorised by age, older imperfect adherers (aged 16-18) saw a statistically significant 10% increase in their adherence ($t(12)=2.50$, $p<0.05$, $d=0.95$). Younger imperfect adherers (aged 13-15) also saw an increase in their adherence (4%), but this was non-statistically significant ($t(26)=0.93$, p≥, $d=0.25$). However, the study was not sufficiently powered for a subgroup analysis.<br><br>**Four sessions (n=21)**<br><br>There was a non-statistically significant increase in medication adherence ($t(14)=-0.529$, $p\geq0.05$), which had a small effect size ($d=0.05$). However, the study was not sufficiently powered for a subgroup analysis.	moderate effect size ($d=0.40$).
Hommel et al. (2011)<br><br>United States of	**Family intervention**<br><br>14 families took part in the intervention. 7	**Intervention group**<br><br>Seven-week intervention with four weekly face-to-face group educational	Pill counts were used to objectively measure the amount of successfully administered	**Pill count.**<br><br>Independent-sample t-tests revealed the intervention group	None.

America.<br><br>RCT.<br><br>Oral medicati on adherenc e	families took part in the intervention, and 7 families took part in the control group.<br><br>No family withdrew from the intervention.	sessions<br><br><u>Session 1:</u> Educational and organisational intervention<br><br><u>Session 2:</u> Behaviour modification<br><br><u>Session 3:</u> Problem solving skills and monitoring adherence<br><br><u>Session 4:</u> Family function<br><br>**Control group**<br><br>Wait list control	medication doses of immunosuppr essants & mesalamine, at baseline and post-intervention.	had statistically significant increases to immunosuppressan ts post intervention compared to the controls (t=2.72, p<0.05). However, the intervention group did not have a statistically significant increase to mesalamine post intervention compared to the controls (t=1.09, p>0.05).<br><br>Paired-sample t-tests examining changes in medication from baseline to post-intervention revealed a non-statistically significant difference across conditions for the intervention and control groups, in adherence rates for immunosuppressan ts (4% increase, t=0.19, p>0.05, d=0.07) and mesalamine (25% increase, t=-1.90, p>0.05, d=0.57).	

Hommel et al. (2012)<br><br>United States of America.<br><br>RCT.<br><br>Oral medication adherence	**Family group intervention**<br><br>20 families took part in the intervention and 20 families were allocated to the control group.<br><br>No families withdrew from the research.	**Intervention group**<br><br>Six-week face-to-face intervention with four weekly educational group sessions. In sessions 1-3 young people and parents met separately. In session 4, young people and parents met together.<br><br><u>Session 1:</u> Educational and organisational intervention<br><br><u>Session 2:</u> Behavioural modification<br><br><u>Session 3:</u> Problem solving skills and monitoring adherence<br><u>Session 4:</u> Family functioning<br><br>**Control group**<br><br>Received usual care	Objective and subjective measures of the amount of successfully administered medication doses were recorded at baseline and post-intervention.<br><br><u>Objective measures</u><br><br>Pill counts and a MEMS track cap was used to record medication adherence behaviours.<br><br><u>Subjective measures</u><br><br>Medication adherence behaviours were recorded using TRAQ	**Pill count***<br><br>Repeated measures ANOVA tests revealed a small non-statistically significant increase (4%) in adherence for those taking immunosuppressants, which was marginally higher than the control group (2%). Results for those taking mesalamine, identified a greater increase in adherence for the intervention group (17%) compared to the control group (6%); this increase was non-statistically significant ($p>0.05$).<br><br>**MEMS track cap***<br><br>Repeated measures ANOVA tests revealed a very small change in adherence for both the intervention group (7%) and the control group (3%), the difference between these two groups were non-statistically significant ($p>0.05$).	None.

				**TRAQ**<br><br>Repeated measures ANOVA tests on parental-reported levels of adherence for immunosuppressants, saw a small non-statistically significant increase in adherence for both the intervention group (8%) and control group (6%). Whereas parental reported adherence rates for mesalamine, identified a non-statistically significant increase in adherence for the intervention group (19%) compared to the control group (2%; $p>0.05$).<br><br>Repeated measures ANOVA tests on patient reported rates for adherence for immunosuppressants also identified a slightly higher rate of adherence for the control group (10%) compared to the intervention group (6%). The difference between these groups were non-statistically significant ($p>0.05$).	

				For mesalamine, the intervention group reported a significant increase (25%) compared to the control group (1%; $p<0.05$).<br><br>F-tests concluded there were a significant increase in mesalamine adherence for the Condition ($F = 22.24$, $p< 0.01$; $d=0.79$) and Condition × Time interaction ($F =13.32$, $p< 0.05$; $d=0.69$).	
Hommel et al. (2013)<br><br>United States of America.<br><br>Single arm clinical trial.<br><br>Oral medication adherence	**Family intervention**<br><br>Nine families were recruited, all of which received the intervention. There was no control group. No families withdrew from the research.	Six-week intervention with four weekly educational sessions conducted over the phone<br><br>Session 1: Educational and organisational intervention<br><br>Session 2: Behaviour modification<br><br>Session 3: Problem solving skills and monitoring adherence<br><br>Session 4: Family functioning	Pill counts were used to objectively measure the amount of successfully administered medication doses of immunosuppressants & mesalamine, at baseline and post-intervention.	**Pill counts.**<br><br>Paired-sample t-tests identified a non-statistically significant increase in adherence rates for immunosuppressants ($t=0.48$, $p>0.05$) and mesalamine ($t=-1.27$, $p>0.05$) from baseline to post-intervention.<br><br>Analysis of the median adherence rates, identified an 8% decrease in adherence rates for immunosuppressants (baseline=61%, post-intervention=53%), which had a small effect size (d=-	None.

				0.17). However, analysis of median adherence rates did show a 29% increase in adherence for mesalamine (baseline: 62%, post-intervention: 91%), which had a large effect size (d=0.63).	
Maddux et al. (2017)<br><br>United States of America.<br><br>Longitudinal single-site noncurrent multiple baseline design across subjects.<br><br>Oral medication adherence	**Family intervention**<br><br>Twelve families were recruited, all of which received the intervention. No families withdrew from the intervention.	Seven-week intervention, with four tailored weekly educational sessions (60-90 minutes long).<br><br>Session 1: Educational and organisational intervention<br>Session 2: Behaviour Modification<br>Session 3: Problem solving skills and adherence monitoring<br><br>Session 4: Family functioning	Pill counts and a MEMS track cap were used to objectively measure the amount of successfully administered medication doses recorded at baseline, post-intervention and at a one month follow up.	**Pill box & MEMS***<br><br>Chi-square analysis identified a statistically significant difference in adherence by timepoint ($p<0.0005$).<br><br>T-tests revealed there was a significant (12%) increase in adherence rates from baseline to post-intervention ($p<0.01$). There was also a significant 6% increase in adherence rates from baseline to the one month follow up ($p<0.05$).<br><br>Odd ratio identified adherence rates at baseline as 2.58 out of 3.58 doses taken. Post-intervention, this increased to 5.09 out of every 6	None.

				doses. At the one month follow up, participants adherence dropped to 3.42 out of every 4.42 doses, however this was still higher than at baseline.<br><br>Logistic regression identified at post-intervention participants had a statistically significant odds ratio when compared to baseline (OR = 1.97, 5.09 2.58, p<0.001). Logistic regression further identified at one-month follow up, participants had a statistically significant odds ratio (OR = 1.36, 3.4/2.58, p<0.0006)	
Vaz et al. (2019).<br><br>USA<br><br>United States of America.<br><br>Oral medicati on adherenc	**Individual intervention**<br><br>Seven young people took part in the intervention and six young people were allocated to the control condition. Two young people withdrew from the intervention and a further two young people withdrew	**Intervention group**<br><br>Received a single 30-minute educational session on gastrointestinal function & anatomy as well as IBD medications.<br><br>**Control group**<br><br>Received usual	Pill dispensers objectively measured the amount of successfully administered medication doses at baseline and post-intervention.<br><br>Post-intervention interviews were conducted to	**Pill dispensers**<br><br>Post-intervention, there was no difference in adherence for the intervention group. There was a non-statistically significant decrease in adherence for the control condition, which had a medium effect size (d=0.85).<br><br>One-month post-	**IBD Knowledge ***<br><br>The interventio n group scored higher on the IBD knowledge questionnai re subscales, however none of these differences

e	from the control condition.	care	assess the feasibility of the intervention and the impact taking part in the intervention had on medication adherence.	intervention, paired-sample t-tests revealed the intervention group had slightly higher adherence (mean=86.04, SD 9.65) compared to baseline. Whereas the control group reported lower adherence (mean=67.1, SD 29.82) compared to baseline. The difference between the groups was not statistically significant (d=0.85, 95% CI [2.51 – 80]).<br><br>**Qualitative Interviews**<br><br>Post-intervention interviews suggested taking part in the intervention did not change participants adherence.	were statistically significant.<br><br>However, within the post-intervention qualitative interviews, participants felt taking part in the intervention did improve their IBD knowledge.<br><br>**Transition readiness questionnaire***<br><br>While mean rank scores showed there were differences between the intervention and control group, none of these were statistically significant

** No effect size reported*

ANOVA, Analysis of Variance; MARS, Medication Adherence Report Scale ; MEMS, Medication Event Monitoring System; PSST, Problem Solving Skills Training; RCT, Randomised Control Trial; TRAQ, Treatment Regimen Adherence Questionnaire; VAS, Visual Analogue Scale.
